# 1254. An Increase in Single-tablet Regimen (STR) Utilization for People Living with HIV (PLWH) Enrolled in Medicaid had Minimal Impact on Pharmacy Costs

**DOI:** 10.1093/ofid/ofac492.1085

**Published:** 2022-12-15

**Authors:** Andrew P Brogan, Cindy Garris, Julie Priest, Victoria Divino, Jing He, Justin Chen, Mitch DeKoven

**Affiliations:** ViiV Healthcare, San Diego, California; ViiV Healthcare, San Diego, California; ViiV Healthcare, San Diego, California; IQVIA, Falls Church, Virginia; IQVIA, Falls Church, Virginia; IQVIA, Falls Church, Virginia; IQVIA, Falls Church, Virginia

## Abstract

**Background:**

The shift to antiretroviral single-tablet regimens (STR) from multiple-tablet regimens (MTR) has lagged for people living with HIV (PLWH) covered by Medicaid. This study examines STR and MTR utilization and pharmacy costs over a 5-year period for PLWH enrolled in Medicaid.

**Methods:**

This retrospective study used IQVIA’s Prescription Claims (Rx) data to identify two mutually exclusive cohorts based on STR or MTR use within each of 5 calendar years (2016-2020). For the STR cohort, the date of the first STR claim in each calendar year was termed the index date. For the MTR cohort, the date of the first MTR drug in the first observed complete MTR regimen in each calendar year was termed the index date; a window of 5 days between prescription fills for the agents used in an MTR regimen was allowed. The regimen received on the index date was used to assign the study cohort for each year and study measures were reported for each of the 5 calendar years. Additional eligibility criteria are provided in Table 1.

**Figure:**
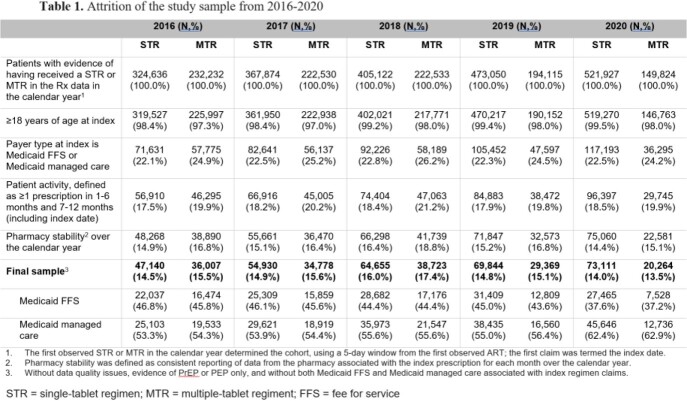

**Results:**

The final STR cohort was 47,140 (14.5% of the initial sample) in 2016 and 73,111 (13.5%) in 2020 (Table 1). The final MTR cohort was 36,007 (15.5%) in 2016 and 20,264 (13.5%) in 2020. The distribution of PLWH with Medicaid Fee-For-Service (FFS) or Medicaid managed care was generally similar by year for both STR and MTR cohorts from 2016 to 2019 (Figure 1); Medicaid managed care enrollment for both cohorts increased in 2020 (62.4-62.9%). Among PLWH, STR use increased annually from 56.7% in 2016 to 78.3% in 2020 (Figure 2). Conversely, MTR use decreased from 43.3% to 21.7% over the same time period. The increase in STR utilization over time was consistent for both plan types. Mean HIV-specific per member per month (PMPM) pharmacy costs were similar across years for both STR and MTR cohorts, ranging from $2,016-$2,342 for STR cohorts and $2,247-$2,380 for MTR cohorts (Figure 3).

**Figure:**
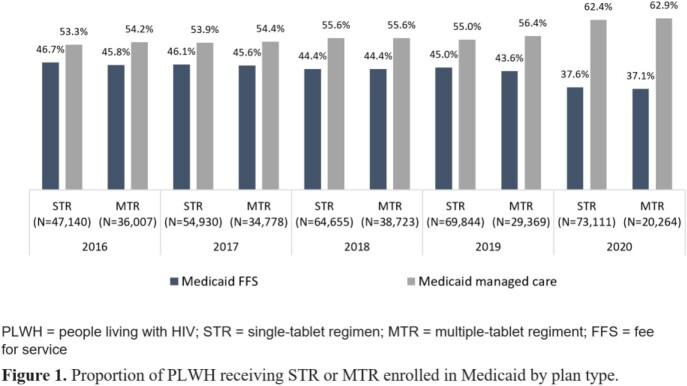

**Figure:**
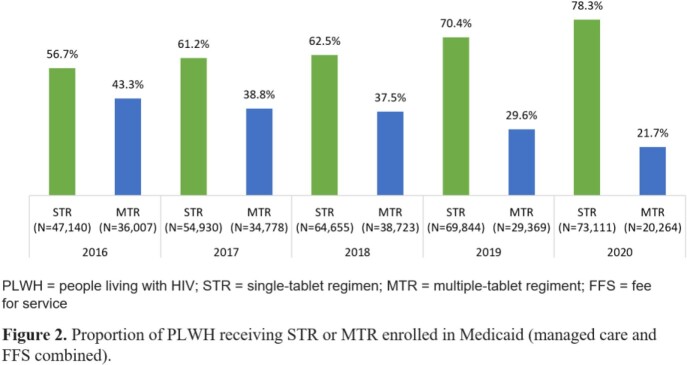

**Figure:**
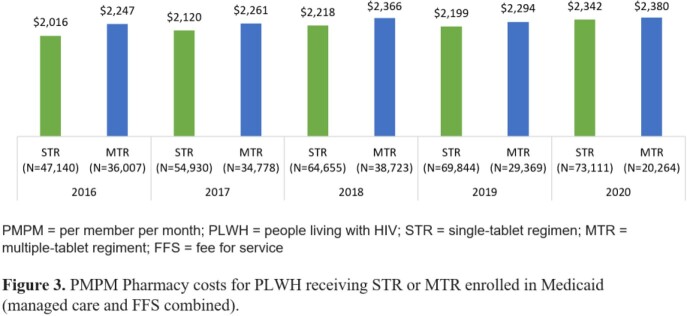

**Conclusion:**

Between 2019 and 2020, PLWH enrolled in Medicaid shifted from FFS towards managed care. STR use among PLWH enrolled in Medicaid increased from 2016 to 2020 with minimal differences in PMPM pharmacy costs compared with PLWH enrolled in Medicaid receiving MTR.

**Disclosures:**

**Andrew P. Brogan, PhD**, ViiV Healthcare: Employee, Salary|ViiV Healthcare: Stocks/Bonds **Cindy Garris, MS**, ViiV Healthcare: Employee|ViiV Healthcare: Stocks/Bonds **Julie Priest, MSPH**, ViiV Healthcare: Employee, Salary|ViiV Healthcare: Stocks/Bonds **Victoria Divino, BA**, IQVIA: Employee, Salary|ViiV Healthcare: Grant/Research Support **Jing He, PhD**, IQVIA: Employee, Salary|ViiV Healthcare: Grant/Research Support **Justin Chen, MHS**, IQVIA: Employee, Salary|ViiV Healthcare: Grant/Research Support **Mitch DeKoven, MHSA**, IQVIA: Employee, Salary|ViiV Healthcare: Grant/Research Support.

